# Surveillance on African Horse Sickness Virus in China’s Southern Border Regions

**DOI:** 10.1155/tbed/6061208

**Published:** 2026-07-09

**Authors:** Feng Zhang, Shujuan Wang, Tiangang Xu, Weijie Ren, Yanli Zou, Yingchao Ren, Yonggang Zhao, Linlin Fang, Shuang Liu, Bangquan Zeng, Min Zheng, Ju Qin, Huanyun Zhao, Qingan Zhou, Jianhao Wu, Amuti Nuermaimaiti, Ronggui Chen, Nan Wang, Jinming Li, Zhicheng Zhang, Zhiliang Wang, Jingyue Bao

**Affiliations:** ^1^ Exotic Herbivore Disease Surveillance and Research Center, China Animal Health and Epidemiology Center, Qingdao, Shandong, China; ^2^ Key Laboratory of Animal Biosafety Risk Prevention and Control(South), Ministry of Agriculture and Rural Affairs, Qingdao, Shandong, China, agri.gov.cn; ^3^ Animal Health Assessment Division, China Animal Health and Epidemiology Center, Qingdao, Shandong, China; ^4^ Exotic Swine Disease Surveillance and Research Center, China Animal Health and Epidemiology Center, Qingdao, Shandong, China; ^5^ Yunnan Center for Animal Disease Control and Prevention, Kunming, Yunnan, China; ^6^ Guangxi Zhuang Autonomous Region Center for Animal Disease Control and Prevention, Nanning, Guangxi Zhuang Autonomous Region, China; ^7^ Xinjiang Uygur Autonomous Region Center for Animal Disease Control and Prevention, Urumqi, Xinjiang Uygur Autonomous Region, China; ^8^ Yili Kazakh Autonomous Prefecture Center for Animal Disease Control and Prevention, Yili Kazakh Autonomous Prefecture, Xinjiang Uygur Autonomous Region, China

## Abstract

African horse sickness (AHS) is a vector‐borne and noncontagious disease caused by African horse sickness virus (AHSV) that poses a severe threat to the global equine industry. As a country historically free from AHS, China faces elevated risks due to recent outbreaks in Southeast Asia in 2020 and its ecological suitability of its border regions for AHSV vectors. Therefore, we conducted consecutive molecular and serological surveillance for AHSV in high‐risk border regions from 2020 to 2024. A total of 3203 equine serum samples, 2909 whole‐blood samples, and 1037 collections of blood‐sucking insects were collected from 103 counties across five border provinces from June to October (peak *Culicoides* season). Samples were analyzed using World Organization for Animal Health (WOAH)‐recommended RT‐qPCR (targeting VP7 gene) and blocking ELISA. Morphological identification confirmed that the collected vectors were primarily *Culicoides* midges, with some mosquitoes. No AHSV nucleic acid or specific antibodies were detected, which is consistent with China’s AHS‐free status. Combined with predictive models and regional risk factors, the likelihood of AHSV occurrence in southern China is “likely.” The study highlights the urgent need for continuous surveillance and risk assessment to safeguard China’s equine industry. Further regional and ecological studies on vectors and animal transportation are also essential for understanding and mitigating the risks associated with AHSV introduction.

## 1. Introduction

African horse sickness (AHS) is an infectious, noncontagious arthropod‐borne disease of equidae caused by African horse sickness virus (AHSV), posing a significant threat to global equine health and the equine industry [1, 2]. AHSV is a double‐stranded RNA virus belonging to the genus *Orbivirus*, family *Reoviridae*. The virion is an unenveloped particle of a size around 70 nm, the genome of which is composed of 10 double‐stranded RNA segments, encoding seven structural proteins (VP1–VP7), most of which have been completely sequenced for AHSV serotypes 4, 6, and 9, and four nonstructural proteins (NS1, NS2, NS3/NS3A, and NS4) [3–5]. The genus *Orbivirus* also includes bluetongue virus (BTV) and epizootic hemorrhagic disease virus (EHDV), which share similar morphological and biochemical properties with distinctive pathological and antigenic properties as well as final host ranges. The biological host for all three diseases is shared, and transmission is primarily mediated by *Culicoides midges*, with other vectors, such as mosquitoes or ticks, may also play a role in the spread of the virus [6–9].

To date, nine antigenically distinct serotypes of AHSV (AHSV1–AHSV9) have been identified [6]. AHS is endemic in most of sub‐Saharan Africa, and all serotypes of AHSV occur in Africa [10]. Historically, only serotypes 4 and 9 AHSV have been found outside of Africa, including the Middle East, Spain, and Portugal, and these outbreaks resulted in a high number of horse deaths and huge economic losses [11–14]. As one of the most lethal equine infectious diseases, AHS recently emerged in Southeast Asia for the first time, resulting in severe consequences. In 2020, an outbreak of AHS caused by serotype 1 AHSV in Thailand resulted in over 500 equine deaths, leading to the suspension of its “AHS‐free country” status by the World Organization for Animal Health (WOAH) World Assembly of Delegates on 27 March 2020 [15, 16]. Subsequently, Malaysia [17] reported an outbreak of AHS in August 2020, resulting in the suspension of its AHS‐free status on 6 August 2020. These outbreaks have caused significant economic losses, disrupted international horse trade, and raised concerns among neighboring countries and the broader Asian equine industry [18, 19]. In response, WOAH has recommended enhanced AHS surveillance in regions adjacent to affected areas.

China is a historically AHS‐free country that was recognized by WOAH in 2014 and now faces an elevated risk of AHSV introduction. Although Thailand does not share a direct border with China, neighboring countries such as Myanmar and Laos experience climatic conditions—hot and humid—similar to those in China’s southwestern provinces. Meanwhile, the presence of AHS vector insects, including *Culicoides imicola*, has been confirmed in Yunnan Province [20]. Furthermore, the importation of subclinically infected equines represents a significant pathway for the introduction of AHSV. For example, the incursion of AHSV into Thailand could be attributed to subclinically infected zebras imported from South Africa [19]. Therefore, the movement of infected equines and the inadvertent introduction of AHSV‐carrying vectors would further heighten the threat of AHS incursion. Vector surveillance is regarded as one of five key components in AHSV early warning system [21]. Given the risk of AHSV introduction, systematic AHS surveillance in China is imperative.

To assess the risk of AHSV introduction and strengthen preparedness in China, we conducted comprehensive surveillance in designated high‐risk regions, including Yunnan, Guangxi, Guangdong, Hainan, and Xinjiang. This study highlights the urgent need for continuous surveillance to safeguard China’s equine industry. Further regional and ecological studies on vectors and animal transportation are also essential for understanding and mitigating the risks associated with AHSV introduction.

## 2. Materials and Methods

### 2.1. Sample Collection and Study Area

China is geographically bordered by 14 countries, among which Vietnam, Laos, Burma, India, Bhutan, Nepal, and Pakistan are in the south and southwest, and Afghanistan, Tajikistan, Kyrgyzstan, Kazakhstan, Mongolia, and Russia are in the west and northwest, and North Korea in the east. A risk‐based surveillance strategy was implemented from 2020 to 2024, focusing on border regions adjacent to countries not officially recognized by the WOAH as free from AHS. Sampling sites were preferably selected within border provinces with higher equine population densities (Guangxi, Yunnan, Guangdong, Hainan, and Xinjiang), based on equine census data from the China Animal Husbandry and Veterinary Yearbook and published provincial livestock surveys [22, 23]. Furthermore, previous studies have confirmed that *Culicoides* midges are widely distributed in China. Prevalent species such as *C. arakawai*, *C. jacobsoni*, and *C. oxystoma* are found in residential and farm areas in the southeastern region, while newly identified species like *C. luteolus* have been detected in southeastern China, particularly along the China–Myanmar border and neighboring areas. Therefore, specific sampling locations were selected based on these vector distribution patterns, in addition to other key risk factors, including proximity to international borders (e.g., Baise and Chongzuo in Guangxi near Vietnam; Honghe, Lincang, and Xishuangbanna in Yunnan near Myanmar and Laos; Ili Kazakh Autonomous Prefecture in Xinjiang near Kazakhstan), local equine population density, and historical data on vector abundance [20, 24–29]. Within these designated sites, a randomized sampling approach was employed. At each location, paired serum and whole blood samples were collected primarily from horses, with a subset obtained from other equids (donkeys and mules). Whole blood samples were collected aseptically from the jugular vein of equines and stored at −80°C for subsequent RNA extraction. Serum samples were separated by centrifugation and stored at −20°C until further use. Vector surveillance was conducted concurrently at selected sites during the annual peak breeding season for local vectors (June to October). Standardized light traps (1–2 per site) were placed near equine ranches and premises and operated overnight from 5 pm to 9 am.

### 2.2. RNA Extraction and Quantitative Reverse‐Transcription PCR (RT‐qPCR)

Viral RNA was extracted from whole‐blood and insect pool samples using a magnetic bead‐based virus DNA/RNA extraction kit (GENFINE, China) following the manual protocol. The vector collections constituted a mixed pool of ~90–100 insects per tube. The entire pool was used for nucleic acid extraction without prior separation. The RNA was eluted in 80 μL of elution buffer and stored at −80°C until use. RT‐qPCR was performed using One Step PrimeScript III RT‐qPCR Mix with UNG, targeting the VP7 gene segment, with the primers and probe adopted from the method recommended by the WOAH [30]. The following thermal cycling protocol was used: reverse transcription at 52°C for 5 min and pre‐denaturation at 95°C for 10 s, followed by 40 cycles of denaturation at 95°C for 5 s and annealing at 60°C for 30 s. RT‐qPCR was performed on the Bio‐Rad CFX96 Real‐Time PCR detection system. Samples with cycle threshold (Ct) values less than 38 were considered positive. In the RT‐qPCR assays, both positive and negative controls were included. The positive control consisted of an AHSV‐4 VP7 plasmid, while DEPC water served as the negative control.

### 2.3. Blocking Enzyme‐Linked Immunosorbent Assay (ELISA)

Serum samples were tested for specific antibodies against AHSV by using a commercial blocking AHS ELISA Kit (INGEZIM AHSV COMPAC PLUS, Ingenasa, Spain) following the manufacturer’s instructions. Briefly, 100 μL of diluted serum samples were added to appropriate wells of an ELISA microplate. The plate was covered and incubated at 37°C for 1 h. After five wash cycles, 100 μL of conjugate was added to each well. The plate was incubated at 37°C for 30 min. Following another wash step, 100 μL of substrate was added to each well, and the plate was further incubated for 10 min at room temperature. The reaction was stopped by adding 100 μL of stop solution to each well, and optical density (OD) at 405 nm was read within 5 min. The blocking percentage (BP) of each sample was calculated applying the following formula: (OD negative control–OD sample)/(OD negative control–OD positive control) ×100. Samples with BP values higher than 50% were considered positive for antibodies to AHSV. Samples with BP values lower than 45% were considered negative for antibodies to AHSV. Samples with BP values between 45% and 50% are considered doubtful, and they must be retested. In the ELISA assays, the positive and negative controls were provided as components of the commercial kit.

### 2.4. Likelihood of AHSV Occurrence in Southern China’s Border Regions

**Figure 1:**
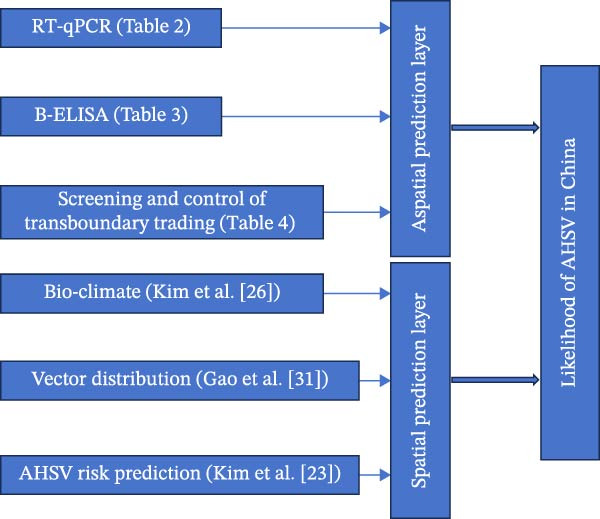
Flowchart of AHSV surveillance and its likelihood occurrence in China.

AHS is a non‐contagious arthropod‐borne disease, the occurrence of its level of likelihood mainly depends on the presence of its vectors, virus, and the infection confirmation of the host animals. By integrating spatial models of bio‐climate determinants, vector distribution, and AHSV risk prediction [26, 27, 31], together with consecutive RT‐qPCR and B‐ELISA results (Table 1, Table 2, Table 3), port‐of‐entry screening of the current status of horse trade between China and neighboring countries, and geographical distribution of Culicoides midges (Table 4), the FAO risk assessment model were employed to assess the result of surveillance (Table 5, Figure 1), and the likelihood of AHSV occurrence in southern China’s border regions was thus generated and concluded.

**Table 1:** Number of samples collected from 2020 to 2024.

Year	Serum	Whole‐blood	Vector (collections)
2020	165	146	17
2021	149	149	476
2022	544	517	123
2023	792	546	93
2024	1553	1551	328

Total	3203	2909	1037

**Table 2:** Results of 3946 equid whole‐blood and vector samples collected from 2020 to 2024 and tested for detection of AHSV nucleic acid using real time RT‐qPCR assays.

Province	2020		2021		2022		2023		2024	
	Number	Result	Number	Result	Number	Result	Number	Result	Number	Result
Guangxi	133	—	204	—	192	—	61	—	456	—
Yunnan	30	—	106	—	430	—	140	—	1386	—
Guangdong	/	/	315	—	18	—	18	—	18	—
Hainan	/	/	/	/	/	/	15	—	19	—
Xinjiang	/	/	/	/	/	/	405	—	/	/

Total	163	—	625	—	640	—	639	—	1879	—

**Table 3:** Results of 3203 equid serum samples collected from 2020 to 2024 and tested for detection of antibodies against AHSV using ELISA.

Province	2020		2021		2022		2023		2024	
	Number	Result	Number	Result	Number	Result	Number	Result	Number	Result
Guangxi	145	—	104	—	214	—	61	—	360	—
Yunnan	20	—	30	—	315	—	239	—	1163	—
Guangdong	/	/	15	—	15	—	15	—	15	—
Hainan	/	/	/	/	/	/	15	—	15	—
Xinjiang	/	/	/	/	/	/	462	—	/	/

Total	165	—	149	—	544	—	792	—	1553	—

**Table 4:** Importation of horses into China from November 2020 to October 2024.

Country/zone of origin of import	Numbers of imported horses
US	62
China HK	80
China Macao	169
Mongolia	180
Argentina	101
Australia	95
Belgium	283
France	119
Ireland	158
Netherlands	3344
New Zealand	254

Total	4845

**Table 5:** Likelihood levels used for the risk assessment of AHSV‐susceptible livestock in unaffected countries/territories being exposed to AHSV (adapted from FAO technical guidelines on rapid risk assessment for animal health threats).

Level of likelihood	Definition
Extremely unlikely	May only occur in exceptional circumstances.
Unlikely	May occur, but not in the majority of instances.
Likely	May occur in the majority of instances.
Very likely	Can be expected to occur frequently.

## 3. Results

### 3.1. Surveillance Design and Sample Collection

AHS is classified as a category I notifiable animal disease in China. Both passive and active surveillance of AHS are implemented annually. For the purpose of identifying risk of AHS infection, a risk‐based sampling strategy was implemented at selected equine ranches/premises adjacent to the international borders in Guangxi, Yunnan, Hainan, Xinjiang, and Guangdong (Figure 2). During the 2020–2024 surveillance period, we collected 3203 equine serum samples, 2909 whole‐blood samples, and 1037 blood‐sucking insect collections from 103 counties in five provinces from June to October (the peak *Culicoides* season) (Table 1). Of the serum samples, 457 were from donkeys and 169 from mules. Similarly, of the whole blood samples, 467 were from donkeys and 169 were from mules. Some blood samples did not clot satisfactorily and were therefore excluded from serological testing. Vectors were collected using standardized light traps deployed in the different surveillance areas. Each collection was sorted, and pools of ~90–100 vector insects, including *Culicoides* (biting midges) and mosquitoes were sent for testing. Based on gross morphological identification, over 90% of the collected insects were *Culicoides* midges, with the remainder being mosquitoes.

Figure 2Origin of the tested samples from 2020 to 2024. Maps show the sampling sites of horses and vectors from 2020 to 2024.

**Figure:**
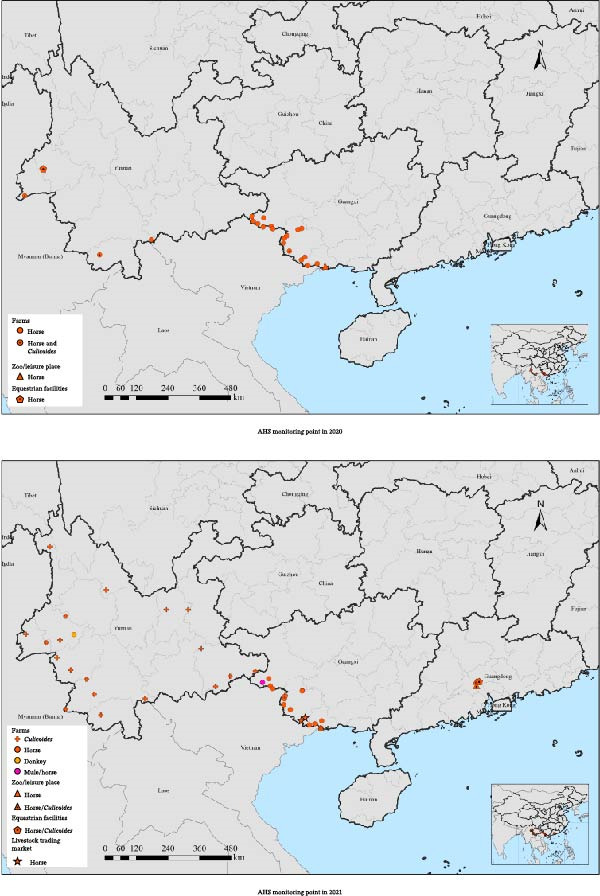

**Figure:**
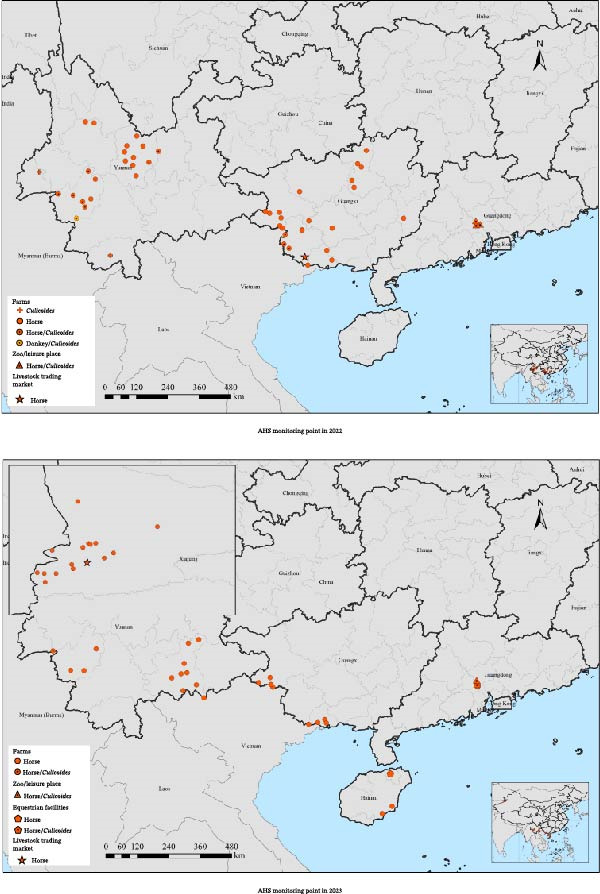

**Figure:**
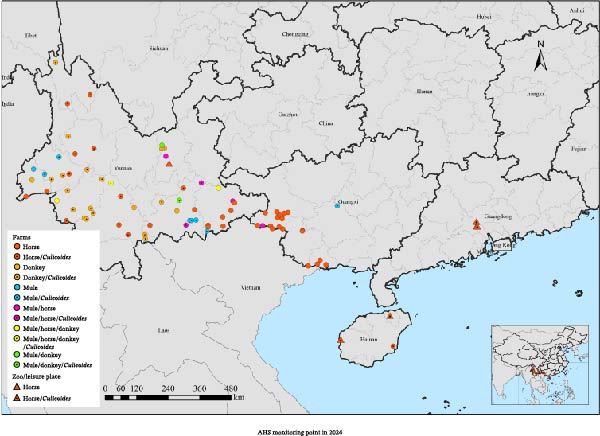

### 3.2. RealTime RT‐qPCR and ELISA Detection of AHSV

Using real‐time RT‐qPCR to detect AHSV nucleic acid targeting the VP7 gene, all whole‐blood and vector samples showed negative results (Table 2). ELISA results revealed that all serum samples were tested negative for AHSV‐specific IgG antibodies (Table 3). These results were consistent with China’s current status as an AHS‐free country.

### 3.3. Likelihood of AHSV Occurrence in Southern China’s Border Regions

Predictive spatial models, which integrated data on bioclimatic variables, *Culicoides* vector distribution, and equine population density, indicated that southern border regions of China possess environmental conditions highly conducive to AHSV persistence and transmission [26, 27, 31]. During the study period (2020 to 2024), extensive field surveillance targeting domestic equines within these modeled high‐risk zones was conducted. All samples tested by RT‐qPCR and B‐ELISA were negative for AHSV nucleic acid and specific antibodies (Tables 2 and 3). In the same period, 4,845 horses were imported from multiple countries and regions, including the US, Belgium, France, Ireland, Mongolia, the Netherlands, Argentina, New Zealand, Australia, *China HK*, and *China Macao*, with no reported AHSV detections in these imported animals (Table 4). In addition, official reports confirm that neighboring Thailand and Malaysia, which previously experienced AHS outbreaks, have now regained their official AHS‐free status. In accordance with the FAO risk assessment framework applied in this study, the combination of environmental suitability, consistently negative surveillance in domestic and imported equines, and the documented return to AHS‐free status in previously affected neighboring countries indicates that the current likelihood of AHSV occurrence in southern China is “likely.”

In Tables 2 and 3: “—” indicates negative test results, and “/” indicates that no samples were collected from that province in the specified year.

## 4. Discussion

AHS is currently an exotic animal disease in China with significant potential for introduction and onward transmission, which could cause devastating outcomes. The research described here represents molecular and serological surveillance of AHSV in equines and vectors across southern China’s border regions. During our 5‐year surveillance of equines and vectors, none of the samples tested positive for AHSV nucleic acids or antibodies. Despite these negative findings, continuous surveillance and further analysis of AHSV occurrence in these regions remain essential for early warning and trend prediction.

The epidemiology of AHSV in Southeast Asia, particularly in Laos and Myanmar, remains poorly documented. Equine populations in these regions are immunologically naïve to AHSV and thus highly susceptible. Therefore, Laos and Myanmar are not recognized as free from AHS according to WOAH Resolution No. 18 (92nd General Session, May 2025). Consequently, we consider these countries to be high‐risk areas. Sampling in regions adjacent to these countries provided highly representative and epidemiologically significant data. A risk‐based surveillance strategy was adopted, focusing on border provinces with long international boundaries and dense equine populations. Given the vast geographic coverage and constraints in personnel and funding, targeted surveillance was implemented rather than a comprehensive census. Sampling sites were selected based on a combination of factors, including proximity to international borders, equine population density, and known vector abundance, followed by random sampling within the chosen counties. Year‐to‐year fluctuations in sample size (Figure 2) primarily reflect variations in funding allocation and field conditions—a recognized limitation in spatial coverage that may introduce bias. Thus, our results confirm the absence of AHSV within the sampled regions only, which is consistent with China’s current AHS‐free status.

It is important to consider the implications of our study’s design for interpreting the negative results within the risk assessment framework. As noted, this investigation was conceived as a targeted, risk‐based survey rather than a statistically powered freedom‐from‐disease study. Consequently, while the consistent absence of AHSV detection across multiple high‐risk compartments over 5 years provides substantial practical evidence against widespread, undetected circulation, it does not constitute formal statistical proof of national or regional freedom. The year‐to‐year fluctuations in sample size and the focused spatial coverage, dictated by resource allocation and field logistics, mean our sampling framework cannot guarantee the detection of a low‐prevalence or focal incursion. However, the core value of this risk‐based surveillance lies precisely in its targeted nature: by concentrating resources on the spatiotemporal and demographic compartments deemed most likely to first reveal virus activity (i.e., border regions with high equine and vector density during peak vector season), it represents a highly efficient strategy for early warning. Therefore, the sustained negative findings, while not absolute, significantly raise the threshold of proof required to assert that a large‐scale, established transmission cycle is currently ongoing undetected. This strengthens the premise that the primary risk is one of future introduction into a receptive environment, rather than one of current, unrecognized endemic transmission.

Given the similarity in insect vectors between BTV and AHSV [32], and because BTV is endemic in some provinces of China, we referenced vector distribution and seasonal data from BTV surveillance [20, 24, 25, 29]. In line with WOAH Terrestrial Code recommendations, the use of a vector surveillance system to detect the presence of circulating viruses (AHSV) is not recommended as a routine procedure because the typically low vector infection rates mean that such detection can be rare. Instead, animal‐based surveillance strategies are preferred [21].

Rapid and sensitive diagnostic tests play a critical role in disease surveillance and international horse trade. In this study, we employed the RT‐qPCR and ELISA methods recommended by the WOAH Manual of Diagnostic Tests and Vaccines for Terrestrial Animals. Compared to conventional RT‐PCR, RT‐qPCR is faster, more sensitive, and versatile [30, 33, 34]. It is an accepted method for AHSV detection and certifying animals for movement, based on foundational work by Agüero et al. and Guthrie et al. [30, 33]. However, there remains some uncertainty in obtaining 100% confidence in negative confirmation. The primary reason for this uncertainty stems from the segmented, double‐stranded RNA (dsRNA) genome of AHSV and its consequent genetic instability, which facilitates rapid evolution and reassortment. This variability can lead to mispriming or probe binding failures, potentially reducing RT‐qPCR accuracy. Guthrie et al. (2013) reported that the median value of diagnostic sensitivity of RT‐qPCR is 97.8% (95% CI: 70.8–99.96%) and the median value of diagnostic specificity is 99.91% (95% CI: 99.3–99.99%). Consequently, the application of this method entails an inherent median false‐negative rate of 2.2% (95% CI: 0.04–30.2%) for AHSV VP7 detection in horses and vector species. A further consideration in interpreting our uniformly negative results is the inherent limitation of any diagnostic assay, namely the potential for false‐negative outcomes. For instance, a comparative evaluation by Penzhorn et al. [35] reported that while the Guthrie et al. RT‐qPCR assay demonstrated 100% sensitivity, the Agüero et al. assay failed to detect the virus in 8.7% of confirmed positive samples, highlighting that assay performance can vary. In this study, we employed the WOAH‐recommended Guthrie et al. assay, which targets the highly conserved VP7 gene and represents the current best available standard for sensitive detection. Nevertheless, the documented possibility of assay failure, however small, necessitates that our conclusion of “no detection” be understood within the context of methodological confidence intervals. This acknowledged limitation does not diminish the value of our large‐scale, consistent negative dataset but underscores the importance of maintaining assay vigilance and periodic validation against circulating strains. Additionally, blocking or competitive ELISA is more specific than indirect ELISA [36–38]. The VP7 blocking ELISA assay employed in this study has been extensively validated. Following the WOAH assay validation pathway, this method has satisfied the requirements for three stages, making it fully adequate for the purposes of this study [39]. Although the methods used in this study are currently the best available choices, close attention should be given to the risk of false negative results.

According to customs statistics, among neighboring countries, only 180 horses were imported from Mongolia during 2020–2024. The importation of donkeys or equine‐derived products from countries with an unknown AHS status requires a specific bilateral protocol, proof of disease‐free status in the exporting country, and strict inspections. Neighboring countries such as Pakistan, Nepal, and India are endemic for BTV, while Vietnam, Laos, and Myanmar have abundant *Culicoides* populations [40]. From a geographical and environmental perspective, China’s southwestern border regions share ecological similarities with Southeast Asian nations that have experienced AHS outbreaks (e.g., Thailand and Malaysia), including climatic conditions favorable to *Culicoides* midges, abundant vector populations, and large equine populations. For an arthropod‐borne disease like AHS, *Culicoides* species play a crucial role in viral transmission and the spread to new previously unaffected areas. Notably, most sampled farms lacked strict biosecurity measures, few had insect‐proof facilities, and insecticide use was limited. Thus, reducing vector‐host contact remains a critical yet challenging aspect of AHSV control.

To reconcile the apparent contradiction between the consistently negative surveillance results and the “likely” risk level assigned, it is crucial to distinguish between the current absence of the virus and the inherent risk of its future introduction and establishment. The “likely” rating, derived from the FAO risk assessment framework, reflects a forward‐looking judgment on the probability of AHSV successfully entering and becoming established in the region, not a statement on its current circulation. Therefore, the conclusion appropriately acknowledges that while the virus is not currently detected, the southern border regions of China possess all the necessary ecological components for sustained transmission if the virus were introduced. This underscores the critical importance of the persistent negative findings: they confirm the ongoing effectiveness of current biosecurity measures but do not diminish the underlying environmental risk that necessitates continued vigilance, enhanced surveillance, and stringent border quarantine protocols.

Consequently, this assessment concludes that the current likelihood of AHSV occurrence in southern China’s border regions is “likely” under the FAO framework. This rating signifies that, although the region is environmentally vulnerable to introduction, the consistent absence of virus detection suggests that AHSV has not yet become established locally. Furthermore, drawing on successful regional control experiences, such as Thailand and Malaysia, which regained AHS‐free status after outbreaks through effective containment, the current risk can be effectively managed through sustained surveillance and early warning, stringent border quarantine measures (particularly for trade with countries that have had previous outbreaks), and enhanced regional coordination in prevention and control. Meanwhile, future systematic surveillance programs should incorporate the necessary sampling intensity and design in accordance with WOAH requirements for risk‐based freedom‐from‐disease analysis so as to enable such assessments to be conducted.

## 5. Conclusion

This study conducted regional AHSV screening to assess the current situation of AHS in southern China’s border regions. All samples tested negative for AHSV by both molecular and serological tests. Based on integrated predictive models and comprehensive surveillance data, the likelihood of AHSV occurrence in southern China is “likely.” These findings not only underscore the urgent need for persistent surveillance but also reinforce the importance of strengthening entry quarantine and border disease prevention measures. Key recommended actions include developing import screening protocols for equids with subclinical infections, establishing sentinel horse herds in high‐risk border zones, and enhancing the monitoring of *Culicoides* vector populations.

## Author Contributions

Jingyue Bao, Zhiliang Wang, and Zhicheng Zhang designed the study. Feng Zhang, Shujuan Wang, Tiangang Xu, Weijie Ren, Yanli Zou, Yingchao Ren, Yonggang Zhao, Linlin Fang, Shuang Liu, Bangquan Zeng, Min Zheng, Ju Qin, Huanyun Zhao, Qingan Zhou, Jianhao Wu, Amuti Nuermaimaiti, Ronggui Chen, Nan Wang, and Jinming Li conducted data collection and analysis. Feng Zhang drafted the manuscript. Jingyue Bao critically revised the manuscript.

## Funding

This work was supported by the National Key Research and Development Program of China (Grant 2022YFD1800500).

## Disclosure

All authors have read and approved the final manuscript.

## Conflicts of Interest

The authors declare no conflicts of interest.

## Data Availability

The data that support the findings of this study are available in this article.
